# Lacosamide Inhibition of Na_V_1.7 Channels Depends on its Interaction With the Voltage Sensor Domain and the Channel Pore

**DOI:** 10.3389/fphar.2021.791740

**Published:** 2021-12-21

**Authors:** Julie I. R. Labau, Matthew Alsaloum, Mark Estacion, Brian Tanaka, Fadia B. Dib-Hajj, Giuseppe Lauria, Hubert J. M. Smeets, Catharina G. Faber, Sulayman Dib-Hajj, Stephen G. Waxman

**Affiliations:** ^1^ Department of Neurology, Yale University School of Medicine, New Haven, CT, United States; ^2^ Center for Neuroscience and Regeneration Research, Yale University, West Haven, CT, United States; ^3^ Rehabilitation Research Center, Veteran Affairs Connecticut Healthcare System, West Haven, CT, United States; ^4^ Department of Toxicogenomics, Clinical Genomics, Maastricht University Medical Centre+, Maastricht, Netherlands; ^5^ School of Mental Health and Neuroscience, Maastricht University, Maastricht, Netherlands; ^6^ Yale Medical Scientist Training Program, Yale School of Medicine, New Haven, CT, United States; ^7^ Interdepartmental Neuroscience Program, Yale School of Medicine, New Haven, CT, United States; ^8^ Neuroalgology Unit, IRCCS Foundation, “Carlo Besta” Neurological Institute, Milan, Italy; ^9^ Department of Medical Biotechnology and Translational Medicine, University of Milan, Milan, Italy; ^10^ Department of Neurology, School of Mental Health and Neuroscience, Maastricht University Medical Center, Maastricht, Netherlands

**Keywords:** voltage-gated sodium channels, local anesthetics, manual and automated electrophysiolgy, voltage-sensing domain, molecular docking

## Abstract

Lacosamide, developed as an anti-epileptic drug, has been used for the treatment of pain. Unlike typical anticonvulsants and local anesthetics which enhance fast-inactivation and bind within the pore of sodium channels, lacosamide enhances slow-inactivation of these channels, suggesting different binding mechanisms and mode of action. It has been reported that lacosamide’s effect on Na_V_1.5 is sensitive to a mutation in the local anesthetic binding site, and that it binds with slow kinetics to the fast-inactivated state of Na_V_1.7. We recently showed that the Na_V_1.7-W1538R mutation in the voltage-sensing domain 4 completely abolishes Na_V_1.7 inhibition by clinically-achievable concentration of lacosamide. Our molecular docking analysis suggests a role for W1538 and pore residues as high affinity binding sites for lacosamide. Aryl sulfonamide sodium channel blockers are also sensitive to substitutions of the W1538 residue but not of pore residues. To elucidate the mechanism by which lacosamide exerts its effects, we used voltage-clamp recordings and show that lacosamide requires an intact local anesthetic binding site to inhibit Na_V_1.7 channels. Additionally, the W1538R mutation does not abrogate local anesthetic lidocaine-induced blockade. We also show that the naturally occurring arginine in Na_V_1.3 (Na_V_1.3-R1560), which corresponds to Na_V_1.7-W1538R, is not sufficient to explain the resistance of Na_V_1.3 to clinically-relevant concentrations of lacosamide. However, the Na_V_1.7-W1538R mutation conferred sensitivity to the Na_V_1.3-selective aryl-sulfonamide blocker ICA-121431. Together, the W1538 residue and an intact local anesthetic site are required for lacosamide’s block of Na_V_1.7 at a clinically-achievable concentration. Moreover, the contribution of W1538 to lacosamide inhibitory effects appears to be isoform-specific.

## 1 Introduction

Chronic pain affects 20–25% of the global population (McCarberg and Billington, 2006; Reid et al., 2011; Kennedy et al., 2014) and is commonly associated with impaired quality of life, opioid addiction, and psychiatric comorbidities (Menefee et al., 2000; Breivik et al., 2006; Ballantyne and LaForge, 2007; Hojsted and Sjogren, 2007; Tunks et al., 2008; Reid et al., 2011). Existing treatments are often limited by inadequate pain relief and severe side effects (Finnerup et al., 2015; Nishikawa and Nomoto, 2017). Pharmacotherapy targeting voltage-gated sodium channels (VGSCs) has been used with some success for the treatment of neuropathic pain (Dib-Hajj et al., 2009; Theile and Cummins, 2011; McKerrall and Sutherlin, 2018; Dib-Hajj and Waxman, 2019; Alsaloum et al., 2020). The anticonvulsant lacosamide (*R*-2-acetamido-*N*-benzyl-3-methoxypropionamide), which is FDA-approved for the treatment of epilepsy, has been investigated as a potential treatment for diabetic neuropathic pain (Shaibani et al., 2009; Wymer et al., 2009; Ziegler et al., 2010), refractory pain (McCleane et al., 2003), and recently, Na_V_1.7-related painful small fiber neuropathy (de Greef et al., 2019), among others (Carona et al., 2021). The clinical effects of lacosamide are thought to be due to its function as a sodium channel blocker (Errington et al., 2006).

Unlike typical antiepileptic drugs (AEDs) such as carbamazepine, phenytoin, and lamotrigine, as well as local anesthetics (LAs), such as lidocaine and benzocaine, lacosamide enhances the voltage-dependence of slow inactivation but not steady-state fast inactivation, and increases use-dependent inhibition of sodium channels (Errington et al., 2008; Sheets et al., 2008; Wang et al., 2011; Niespodziany et al., 2013; Rogawski et al., 2015). This suggested that lacosamide exerts its effect on VGSCs by a different mechanism than AEDs and LAs and Jo and Bean reported slow binding of lacosamide to the fast-inactivated state (Jo and Bean, 2017). Also, LAs and conventional AEDs are pore-blockers that share a common binding motif, the “LA binding site”, composed of residues in the S6 segment of domains DI, DII, and DIV (Ragsdale et al., 1996; Liu et al., 2003; Nau and Wang, 2004; Lipkind and Fozzard, 2005, 2010; Yang et al., 2010), which include the critical residues phenylalanine (F1764, Na_V_1.7 numbering) and tyrosine (Y1771) (Ragsdale et al., 1996; Kuo, 1998). Previous studies have posited that lacosamide’s binding site is within the permeation pathway, overlapping with the binding site for batrachotoxins and LAs in the S6 helix (Stevens et al., 2011; Wang and Wang, 2014; Jo and Bean, 2017); however, radio-ligands essays have failed to assign a specific residue to lacosamide binding against hundreds of known receptors and binding sites (Errington et al., 2006). These data suggest a complex mechanism for lacosamide binding and inhibition of VGSC.

The Na_V_1.7-W1538R mutation, located in the S2 helix of the voltage sensing domain of the channel’s domain IV (VSD4), causes a hyperpolarizing shift in the voltage-dependence of activation of Na_V_1.7 channels and has been implicated in the pathology of both inherited erythromelalgia (Cregg et al., 2013) and small fiber neuropathy (Eijkenboom et al., 2019). Furthermore, the mutation has been shown to abolish the effect of lacosamide on slow-inactivation and use-dependent inhibition of the channel at clinically-achievable concentrations (Labau et al., 2020)**.** Interestingly, an arginine residue is a natural variant at the corresponding position to W1538 in Na_V_1.1 and Na_V_1.3 channels (R1560 in human Na_V_1.3), and Na_V_1.3 has been shown to be less sensitive to lacosamide, compared to Na_V_1.7 (Sheets et al., 2008). Importantly, the W1538 residue is one of three residues required for selective blockade of Na_V_1.7 by aryl sulfonamide small molecules (McCormack et al., 2013; Jo and Bean, 2020). Aryl sulfonamides are nanomolar potent sodium channel inhibitors, which exhibit up to 1000-fold selectivity for specific channel subtypes. Similar to lacosamide, they show a slow onset of block (Theile et al., 2016). Moreover, these compounds exhibit binding to the extracellular surface of the VSD4, independently of an intact LA binding site in the pore (McCormack et al., 2013; Jo and Bean, 2020). These data suggest that lacosamide inhibition of Na_V_1.7 may share mechanistic features with AEDs/LAs and aryl-sulfonamides.

In this study, we show using voltage-clamp analysis that lacosamide requires both an intact LA binding site in the pore and the W1538 residue in VSD4 for effective Na_V_1.7 inhibition by a therapeutic concentration of lacosamide. Using molecular docking analysis of human Na_V_1.7 channels, we propose that W1538 is a putative high affinity site, which possibly guides lacosamide to the pore. In support of this view, high doses of lacosamide block Na_V_1.7-W1538R mutant channels. Additionally, we demonstrate that the W1538R mutation is sufficient to render Na_V_1.7 channels sensitive to the Na_V_1.3-selective aryl sulfonamide blocker ICA-121431. We also show that the reciprocal mutation (R1560W) in Na_V_1.3 was insufficient to render Na_V_1.3 channels sensitive to lacosamide at clinically-achievable concentrations, suggesting that the effect of the tryptophan residue in VSD4 on lacosamide block is isoform-dependent.

## 2 Methods

### 2.1 Plasmids and Cell Culture

The constructs for wild-type (WT) carrying the adult-long (AL) splicing isoforms of the human Na_V_1.7 and Na_V_1.3 channels were made tetrodotoxin-resistant (TTX-R) by Y362S and Y384S substitutions, respectively. A green fluorescent protein (GFP)-2A linker was then fused in-frame at the N-terminus of the channel, as previously described (Yang et al., 2016). The GFP-2A-Na_V_ construct allows the production of separate GFP and Na_V_ proteins from the same transcript, thus enabling the visual identification of the channel-expressing cells by green fluorescence labeling. Mutations in these WT channels were introduced by site-directed mutagenesis using QuickChange XL (Stratagene, La Jolla, CA, United States), and identity of the inserts was confirmed by Sanger sequencing. The following mutations were introduced into the Na_V_1.7 and Na_V_1.3 WT channels: Na_V_1.7-F1737A/Y1744A mutant (established to prevent the binding of LAs and AEDs in the pore (Ragsdale et al., 1996; Liu et al., 2003; Panigel and Cook, 2011; McCormack et al., 2013), hNa_V_1.7 -W1538R; and hNa_V_1.3 2A-R1560W. Constructs were transfected into human embryonic kidney (HEK293) cells (1 μg/μL) using Lipojet (SignaGen Laboratories) for transient expression together with β1 and β2 subunits: pCD8-IRES-hB1 (0.5 μg/μL) and pCDNA3-hB2 (0.5 μg/μL).

HEK293 cells were grown in DMEM/F12 (Dulbecco’s modified Eagle’s medium), supplemented with 10% fetal bovine serum and 1% penicillin/streptomycin, and incubated at 5% CO2 and 37°C. The cells were passaged 1–2 times per week and media was replaced 1 hour before transfection. Transfected cells were resuspended in TryplE Express (ThermoFischer) and either replated onto laminin/PDL-coated coverslips for manual voltage patch-clamp recordings or resuspended in Sophion extracellular bath solution for automated voltage-clamp recordings on the Qube 384 (Sophion Bioscience, Inc.) (Qian et al., 2020).

### 2.2 *In vitro* Pharmacology—Reagents

The effect of lacosamide, obtained from either Adooq (Adooq Biosciences, A10510) or UCB (Vimpat®, West Haven VA Medical Center pharmacy), was assessed both at a clinically achievable concentration (30 μM), based on the maximum plasma concentration of patients receiving a daily dose of 200 mg (Cawello, 2015; de Greef et al., 2019), and at 300 μM. At a concentration of 10 mg/ml (39.9 mM) in saline (pH 4), Vimpat was diluted in extracellular bath solution to achieve a final concentration of either 30 μM or 300 μM. The formulation was used to evaluate the effect of therapeutic lacosamide concentration (30 μM) and 10-fold that dose (300 μM) on Na_V_1.7-W1538R and Na_V_1.3-R1560W mutant channel properties.

Lacosamide from Adooq Bioscience was used to test the drug on the Na_V_1.7-F1737A/Y1744A channels. The compound was diluted in dimethyl sulfoxide (DMSO; Tocris Bioscience, Cat. No. 3176) at 100 mg/1.33 ml to give a 300 mM stock solution. Further dilutions were performed to a working solution of 30 μM in 0.1% DMSO in extracellular bath solution.

Lidocaine (lidocaine hydrochloride monohydrate, Sigma Aldrich, L5647) was diluted in Sophion extracellular bath solution, as described (Qian et al., 2020), to make a stock solution of 80 mM, and then diluted 1,000x to a working concentration of 80 μM in extracellular bath solution.

The aryl sulfonamide Na_V_1.3-selective blocker ICA-121431 was obtained from Adooq Bioscience (A13773). ICA-121431 was first solubilized in DMSO to make a stock solution of 50 mM. The stock was diluted with DMSO to a 0.1 mM concentration, before further dilution to a final concentration of 0.1 μM in 0.1% DMSO in Sophion working extracellular solution (Qian et al., 2020). Dosage was selected based on prior publication (McCormack et al., 2013) and preliminary dose-response assay using 0.01, 0.1, and 1 μM (*data not shown*).

The stock solutions were kept in the freezer at −20°C; working solutions were prepared fresh daily and kept at room temperature (RT).

### 2.3 Electrophysiology

#### 2.3.1 Manual Voltage-Clamp Recordings

Biophysical responses of Na_V_1.7 sodium channels to different treatments were evaluated by whole-cell voltage-clamp analysis. The recordings were performed at RT on isolated GFP-positive HEK293 cells, alternating between cells expressing a mutant and its corresponding WT channel on the same day. Furthermore, each pair recorded was assessed with either vehicle or treatment on one given day. As such, the Na_V_1.7-WT and W1538R pair were recorded with either 30 μM or 300 μM lacosamide and vehicle (extracellular bath) on the same day, and the alternative concentration, on a separate day. For each coverslip, a single cell was recorded, either exposed to treatment or vehicle.

##### 2.3.1.1 Solutions

The vehicle, or extracellular bath solution, contained (in mM): 140 NaCl, 3 KCl, 1 MgCl2, 1 CaCl2, 10 HEPES. The pH was adjusted to 7.3 with NaOH, and the solution was brought to 320–330 mOsm using dextrose.

The intracellular solution contained (in mM): 140 CsF, 10 NaCl, 10 HEPES, 1 EGTA. The pH was adjusted to 7.3 with CsOH. The solution was brought to 310–320 mOsm with dextrose, before being used to fill borosilicate glass micropipettes (WPI; 1.65-mm outer diameter) containing the recording electrode. The pipettes were pulled to a 0.7–1.5 MΩ resistance to maintain a series resistance under 4 MΩ.

##### 2.3.1.2 Protocols

Initiation of the voltage-clamp protocols was set at 5 min following breakage of the cell membrane. All recordings were obtained with whole-cell configuration using an EPC-10 USB amplifier (HEKA Electronics), with the software PatchMaster (HEKA Electronics). Data was acquired at a rate of 50 kHz, with a low-pass 2.9 kHz Bessel filter.

To minimize voltage errors, the series resistance was compensated to 40–90%, and only cells that could maintain a voltage error below 3.5 mV were analyzed. Linear leak currents, along with capacitance artefacts were corrected, whenever appropriate, using a P/6 subtraction.

The voltage-clamp protocols were systematically applied in the following order to reduce the risk for time-dependent variation in treatment effects:

Activation was evaluated by applying a 5 mV series of depolarizing increments for 100 ms, starting from −80 mV to +50 mV, every 5 s, from a −120 mV holding potential.

Steady-state fast-inactivation was measured by holding the membrane potential for 500 ms at conditioning potentials, varying from −140 to 10 mV, with 10 mV depolarizing increments.

Use-dependent block of Na_V_1.7 channels with lacosamide was measured at 20 Hz using a series of 20 ms pulses applied at −10 mV and following a −120 mV holding potential. The peak inward currents were normalized to their maximum current amplitude.

Slow-inactivation was evaluated by holding the cell for 30 s at conditioning potentials ranging from −130 to 10 mV (with 10 mV increments), after which a -120 mV pulse was applied to the membrane potential for 100 ms to enable any channel not in the slow-inactivated state to recover from fast-inactivation. After both (fast and slow) inactivation protocols, an additional 20 ms test pulse at 0 mV was used to elicit current from any remaining available channels.

##### 2.3.1.3 Perfusion

Vehicle and lacosamide were perfused at a continuous flow rate of 1 ml/min, 2 min after establishing whole-cell configuration and for two uninterrupted minutes. Alternatively, cells recorded in larger chambers were perfused at a continuous flow rate of 0.3 ml/min, 2 min after break-in and for 8-9 uninterrupted minutes or until the initiation of the slow-inactivation protocol. Complete bath exchange was ensured by using a pressure-regulated system which allowed consistent solution distribution on one end of the chamber (AutoMate Scientific), through a 250 μm perfusion pipette, and by slowly aspirating it from the opposite end, as previously described (Labau et al., 2020).

To control for time-dependent and perfusion-related pressure-dependent alterations in channel properties, each cell was exposed consistently to only one solution at a time and for each recording.

#### 2.3.2 Automated High-Throughput Patch-Clamping: Qube 384

The Qube automated electrophysiology instrument from Sophion Bioscience was utilized to perform higher throughput assessment of the effects of lidocaine, lacosamide and ICA-121431. A detailed description of the capabilities and the setting up of modules to perform voltage-step protocols for the Qube instrument has previously been published (Qian et al., 2020). In general, the pulse protocols were configured to match as closely as possible those used in the manual patch-clamp experiments. There were two notable differences when performing our experiments on the Qube compared to manual patch-clamp. The first was that the Qube performs a solution exchange by utilizing a pipetting step which dispenses 7 μL per well. The volume of the part of the well that contains the recorded cell is approximately 1 μL, so a solution change results in a seven-fold volume exchange. The second difference was the implementation of leak subtraction and series resistance compensation. The Sophion instrument does not implement P/n pulses but instead performs an analysis of the transient response to small test pulses which are then scaled to match the applied pulses resulting in leak-subtracted trace data. For series resistance compensation, Sophion has implemented a method developed by Alembic (Sherman et al., 1999), which is less prone to positive feedback oscillations and can reach 100% compensation. For the data presented here, the series resistance compensation level was set to 90%.

##### 2.3.2.1 Solutions

The solutions used when performing experiments with the Qube are as recommended by Sophion (Qian et al., 2020). Briefly, the standard extracellular solution contained (in mM): NaCl (145), KCl (4), CaCl2·(2H_2_O; 2), MgCl2·(6H_2_O; 1), HEPES (10) titrated to pH 7.4 with NaOH and osmolarity was adjusted to 305 mOsm with glucose.

The standard intracellular solution contained (in mM): CsF (140), EGTA (1), HEPES (10), NaCl (10), titrated to pH 7.3 with CsOH and osmolarity was adjusted to 310 mOsm with glucose. Compounds or vehicle were diluted from stock solutions into Sophion standard extracellular solution and placed into compound plates of various formats, where we tested vehicle against up to two different compounds at different dilutions (or a minimum of one solution in 32 wells per genotype per plate), and with each cell exposed to a different compound. For lidocaine, however, we implemented a “before-and-after recording” system, where each cell was first exposed to vehicle (extracellular solution) and then to 80 µM lidocaine.

##### 2.3.2.2 Protocols

The voltage-clamp pulse protocols were implemented on the Qube instrument so as to replicate the ones used for manual patch-clamp. Shortly after establishing the whole-cell configuration, the cells were pulsed from a holding potential of −120 mV to a 0 mV stepping voltage for 50 msec, after which, the voltage was returned to the holding potential and the step was repeated with a 10 s interval. The whole protocol was repeated for 1 minute, after which, the pipetting solution exchange was initiated. Three complete solution exchanges were implemented at 1 min intervals during the recurring pulses. Following each well equilibration with its designated compound and concentration, the biophysical characterization was performed. Similar to manual patch-clamp experiments, protocols were applied in a specific order to prevent time-dependent bias, as follows:

Voltage-dependence of activation consisted of 100 ms duration pulses, from a holding potential of −120 mV, that were stepped from −80 mV, then returned to the original holding potential. Step depolarization was applied in 5 mV increments and looping at 5 s intervals until the last pulse to 10 mV.

Voltage-dependence of fast-inactivation was measured by holding the cell at −120 mV, after which conditioning pulses of 500 ms were applied from −120 to 20 mV in 10 mV increments. Following the conditioning pulse, a test pulse to 0 mV was applied for 50 ms to determine the fraction of sodium channels still available for opening. The potential is then returned to the holding potential of −120 mV until the next loop occurring at 5 s intervals.

Use-dependence of inhibition consisted of a train of 20 ms 20 Hz depolarizing pulses to 0 mV, starting from a holding potential of −120 mV.

Voltage-dependence of slow-inactivation consisted of 20 s long pulses starting at −140 mV and stepping with 10 mV increments until the last loop at 20 mV. Each loop was spaced by 30 s. Following the conditioning pulse, a brief 100 ms pulse to −120 mV was used to recover the remaining channels from the fast-inactivated state, which was then stimulated by a 100 ms pulse to 0 mV prior to returning to the initial holding potential of -120 mV. The peak inward currents were normalized to their maximum current amplitude. Cells that were selected for analysis were those that had a peak series resistance < 20 MΩ, average membrane resistance > 200 MΩ, a peak current > 1 nA, and a fit quality (as determined by the Sophion Boltzmann fit of the data) < 0.15 for activation and 0.05 for fast-inactivation and slow-inactivation, as well as minimal leak (<10% of the peak sodium current).

### 2.4 Structural Modeling and Molecular Docking

The Protein Data Bank (PDB) structure of the Na_V_1.7 channel (PDB ID: 6J8I) from (Shen et al., 2019) was edited to remove all toxins and auxiliary subunits. Blind docking analysis utilizing the Achilles Blind Docking server was subsequently performed for lacosamide (ZINC structure ZINC7673) (Sterling and Irwin, 2015) on the Na_V_1.7 α-subunit. The channel was visualized using PyMol (Schrödinger, LLC).

### 2.5 Statistical Analyses

Manual electrophysiology data were obtained with FitMaster (HEKA Electronics) and automated electrophysiology data were obtained with Sophion Analyzer v6.5.76 (Sophion Instruments).

All datasets were analyzed in Excel (Microsoft), OriginLab (OriginLab Corporation, Microcal Software, Northhampton, MA) and/or GraphPad Prism 8.4.3.

Data are expressed as means ± standard error of the mean (SEM). Statistical significance was determined by *p < 0.05 and **p < 0.01 using either unpaired Student’s t-tests or One-way ANOVA. When ANOVA was used, Dunnett’s multiple comparison analysis was run for the means to assess significant differences between the treatment groups and control.

Voltage-dependent activation was analyzed as the conductance (G), calculated using the following equation: G = I/(V-V_rev_), where I is the peak current at each voltage (V) measured, and V_rev_ is the reversal voltage calculated by extrapolating peak currents with depolarizing potentials ranging from 10 to 40 mV. Conductance was normalized to the maximal sodium conductance (G_max_) for a given cell, and curves were fitted using the Boltzmann equation: G/G_max_ = 1/{1 + exp [(V_1/2_-V)/*k*]}, where V_1/2_ is half the maximal activation voltage, and *k* the slope factor. Steady-state fast and slow inactivation residual currents were also normalized to the maximal sodium current (I_max_), plotted against the voltage of incremental conditioning pulses. The inactivation curves were fitted with the Boltzmann equation, as follows: I/I_max_ = 1/{1 + exp [(V_1/2_-V)/*k*]}, when appropriate. Alternatively, when the curves had two distinct components, a double Boltzmann equation was applied: I/Imax = 1/{F [1 + exp (V-V1_1/2_)/k_1_]}+{1-F [1 + exp (V-V2_1/2_)/k_2_]}, where F is the first component’s fraction and 1-F is the second component’s fraction. For use-dependence of inhibition, the peak current of each evoked pulse was normalized to the first pulse of the series and significance was evaluated by comparing the control’s last pulse mean to those of the drug groups.

## 3 Results

### 3.1 The W1538 Region is Predicted as the Most Energetically Favorable Binding Site for Lacosamide

**FIGURE 1:**
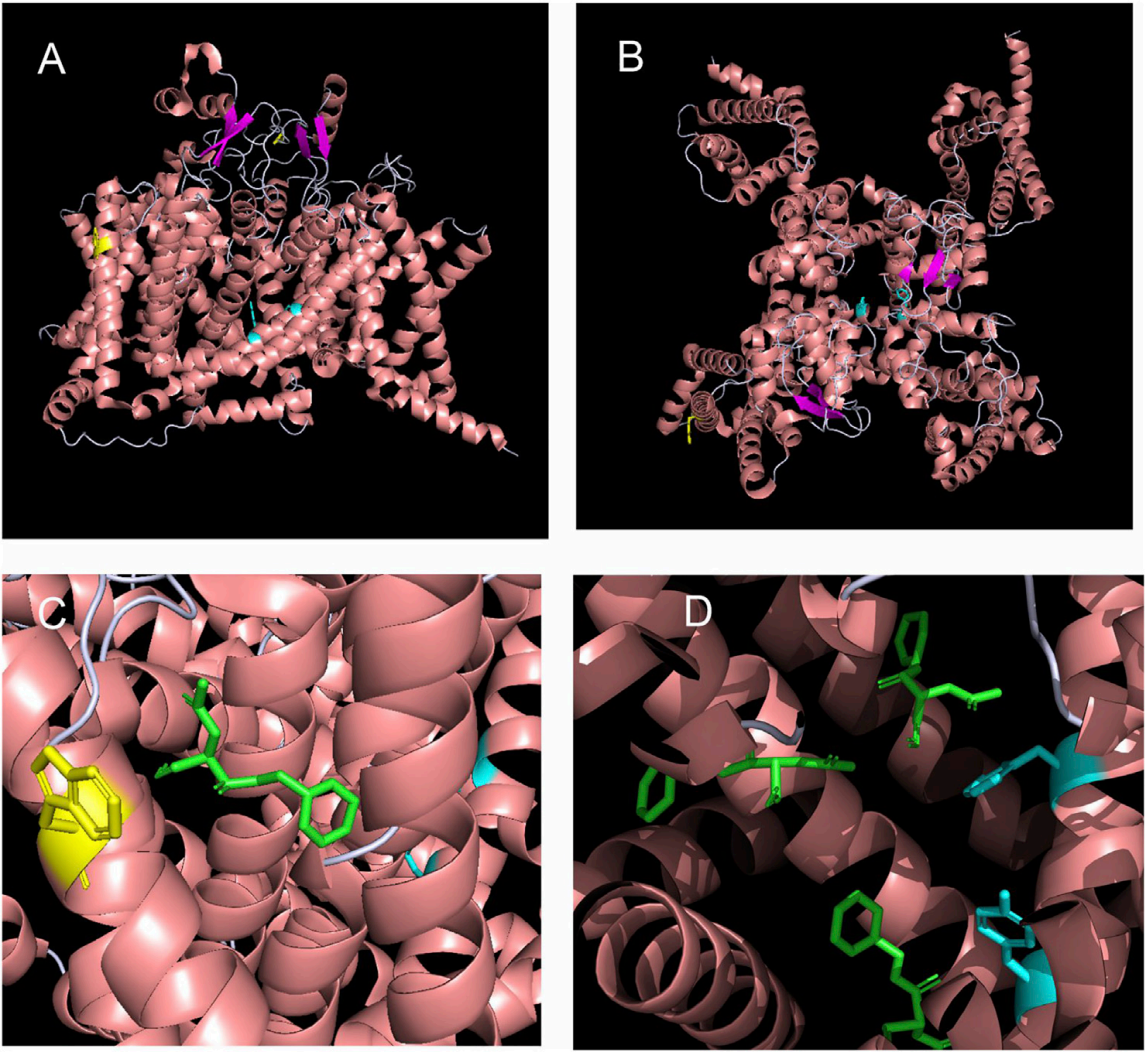
Structural modeling and unbiased docking simulations suggest binding sites of lacosamide on Na_V_1.7 channels. **(A,B)** Structural analysis of sodium channels allows the visualization of the W1538 residue in the VSD of DIV, as shown in yellow. The local anesthetic (LA) site F1737 and Y1744 residues locate in the pore of the channel and are both shown in cyan. Structure of the Na_V_1.7 voltage-gated sodium channel is based on Shen et al. (2019) and is represented **(A)** from the side and **(B)** from the top. **(C,D)** *In silico* docking identifies the LA site (F1737/Y1744) and the W1538 as putative binding sites of lacosamide on Na_V_1.7 sodium channels. **(C)** Lacosamide (green) docked in its most favorable position is predicted by blind docking. The W1538 side chain is shown in yellow. **(D)** Lacosamide (green) docked in its second, fourth, and eighth most favorable positions, predicted by blind docking, near the LA binding site.

Neither the binding characteristics of lacosamide to Na_V_1.7 nor the underlying mechanisms of action by which it blocks the channel are well understood. To address this gap in knowledge and assess possible allosteric effect of lacosamide on the pore and determine potential structural mechanisms underpinning lacosamide’s mode of action, we performed unbiased docking analysis for lacosamide on the Na_V_1.7 α-subunit (Figure 1). This method allows us to predict the preferred binding sites for the compound with no bias towards particular local structures of the channel. This analysis identified 1,139 possible poses, which could be grouped into 62 distinct clusters with non-overlapping coordinates. The top 10 most energetically favorable clusters had binding energies more negative than −6.2 kcal/mol. The most energetically favorable cluster for lacosamide binding docked the compound near the W1538 residue, with the most energetically favorable pose in this cluster approximately 8.8 Å away, although it shared no discernible interactions directly with the W1538 residue (Figure 1C). The binding energy for lacosamide at this site was approximately −7.3 kcal/mol. Interestingly, the second, fourth, and eighth most energetically favorable clusters for lacosamide docking were found near the F1737 and Y1744 residues (Figure 1D). Thus, while the most energetically favorable site for lacosamide binding is near to W1538, lacosamide also readily binds in many positions around the LA binding site.

### 3.2 Lacosamide Fails to Exert its Inhibitory Effects on the Na_V_1.7-F1737A/Y1744A Mutant

**FIGURE 2:**
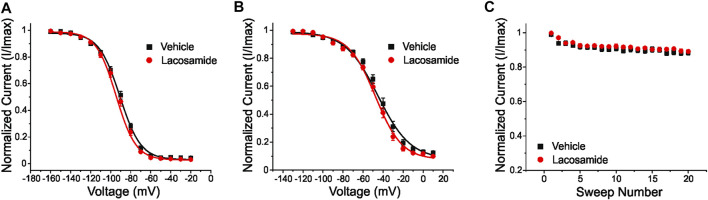
The local anesthetic binding site (F1737/Y1744) in the channel pore is necessary for inhibition of Na_V_1.7 by lacosamide. The clinically-achievable concentration (30 μM) of lacosamide does not block Na_V_1.7 channels with a disabled LA binding site (F1737A/Y1744A). **(A)** No difference in the voltage-dependence of steady-state fast-inactivation was observed with the application of 30 μM lacosamide in mutant Na_V_1.7 channels lacking a functional LA binding site. **(B)** Similarly, the voltage-dependence of slow-inactivation was unchanged. **(C)** Abolishing the LA binding site resulted in no appreciable effects of lacosamide on Na_V_1.7 use-dependent inhibition at 20 Hz.

Having shown that lacosamide exhibits high affinity binding near both the W1538 residue and the LA binding site, we next sought to investigate whether lacosamide does indeed require active binding to the pore to block VGSCs. We introduced the F1737A/Y1744A mutation into Na_V_1.7 channels using site-directed mutagenesis, abolishing the LA binding site (McCormack et al., 2013; Panigel and Cook, 2011). Mutated constructs were transfected into HEK293 cells for transient expression. Whereas lacosamide readily inhibits Na_V_1.7-WT channels at the therapeutic dose of 30 μM (Errington et al., 2008; Labau et al., 2020), it did not cause a hyperpolarizing shift in the voltage-dependence of slow-inactivation in the F1737A/Y1744A mutant channels (Figure 2). Using unpaired Student’s t-tests, the voltage of half maximal slow inactivation (V_1/2_; Figure 2B) of the mutant channel after treatment with lacosamide was statistically comparable to channels treated with vehicle (0.1% DMSO; Table 1). Furthermore, lacosamide’s known enhancement of use-dependent block of the Na_V_1.7-WT channel (Labau et al., 2020), measured by a series of twenty 20 Hz pulses, was lost in Na_V_1.7-F1737A/Y1744A mutant channels (Figure 2C; Table 1), indicating that, at clinically-achievable concentrations, lacosamide requires a functional LA binding site to affect Na_V_1.7 function. Finally, lacosamide did not alter the V_1/2_ of steady-state fast-inactivation in F1737A/Y1744A-expressing cells (−92.5 ± 1.5 mV, *n* = 12, Figure 2A), compared to vehicle (−91.3 ± 1.3 mV, *n* = 13, *p* = 0.51).

**TABLE 1:** Effects of lacosamide (LCM) at the clinically-achievable concentration of 30 μM and at 300 μM on NaV1.7 and NaV1.3 wild-type and mutant channels. Lacosamide significantly enhanced slow inactivation and use-dependence of inhibition in Na_V_1.7-WT channels at both 30 and 300 μM compared to vehicle, Vh.1 for 30 μM and Vh.2 for 300 μM (Vehicle; extracellular solution), as per different recording days for manual patch clamp analysis. Clinically-achievable concentrations (30 μM) of lacosamide had no effect on any of the mutant channels (ie. Na_V_1.7-W1538R, Na_V_1.7-F1737A/Y1744A and Na_V_1.3-R1560W) or Na_V_1.3-WT channels. However, at the higher dose of 300 μM, lacosamide evoked a significant hyperpolarizing shift in slow inactivation of Na_V_1.3-WT, Na_V_1.3-R1560W and Na_V_1.7-W1538R mutant channels. In both Na_V_1.7-WT and W1538R channels, 300 μM lacosamide-induced slow inactivation hyperpolarizing shift was best fitted by a double Boltzmann, therefore yielding two half-inactivation (V_1/2_) voltages. Here, we only report the V_1/2_ of the first component occurring during the first drug response phase and corresponding to the V_1/2_ of most inactivated channels. Na_V_1.3-WT and R1560W channels were fitted with a single Boltzmann. A 10-fold increase in the lacosamide therapeutic dose also significantly enhanced use-dependent block in Na_V_1.7-W1538R mutant channels, but not in any of the Na_V_1.3 variants. The effect of 300 μM lacosamide was not tested in Na_V_1.7- F1737A/Y1744A channels. The Qube allowed the same vehicle group to be tested against Na_V_1.3 variants, where data from vehicle 1 = vehicle 2. Data are presented as Means ± SEM.

			Na_V_1.7-WT	Na_V_1.7- W1538R		Na_V_1.7- F1737A/Y1744A	Na_V_1.3-WT	Na_V_1.3-R1560W
Lacosamide	Slow inactivation V_1/2_ (mV)	Vh.1	−63.47 ± 1.75, *n* = 13	−71.24 ± 2.68, *n* = 6	−43.1 ± 2.2, *n* = 13		−51.45 ± 0.92, *n* = 27	−52.17 ± 1.08, *n* = 27
		LCM 30 μM	−71.12 ± 1.86, *n* = 11, *p* = 0.007	−74.61 ± 2.22, *n* = 8, *p* = 0.35	−47.1 ± 1.6, *n* = 12, *p* = 0.16		−54.78 ± 1.87, *n* = 17, *p* = 0.4	54.50 ± 1.41, *n* = 32, *p* = 0.47
		Vh.2	−77.56 ± 2.5, *n* = 5	−79.36 ± 2.04, *n* = 5	—		*= Vehicle 1*	*= Vehicle 1*
		LCM	−113.71 ± 0.22	−117.32 ± 1.99	—		−62.95 ± 2.9, *n* = 21, *p* < 0.0001	−58.32 ± 1.94, *n* = 32, *p* = 0.014
		300 μM	*N* = 6	*N* = 6						
		—	*p* = 1.25e^−6^	*p* = 7.05e^−5^						
	Use-dependent block	Vh.1	0.9 ± 0.01, *n* = 10	0.91 ± 0.01, *n* = 6	0.88 ± 0.01, *n* = 13		0.88 ± 0.01, *n* = 21	0.87 ± 0.05 *n* = 27
		LCM	0.86 ± 0.02, *n* = 7, *p* = 0.04	0.88 ± 0.02, *n* = 8, *p* = 0.30	0.89 ± 0.01, *n* = 13, *p* = 0.68		0.87 ± 0.01, *n* = 18, *p* = 0.5	0.88 ± 0.05, *n* = 27, *p* = 0.61
		30 μM								
		Vh.2	0.8 ± 0.03, *n* = 5	0.85 ± 0.02, *n* = 5	—		*= Vehicle 1*	*= Vehicle 1*
		LCM	0.69 ± 0.02, *n* = 6, *p* = 0.0083	0.67 ± 0.06, *n* = 6, *p* = 0.033	—		0.85 ± 0.01, *n* = 29, *p* = 0.056	0.83 ± 0.08, *n* = 32, *p* = 0.07
		300 μM								

### 3.3 The W1538 Residue is not Critical for Lidocaine Binding to Na_V_1.7 Channels

**FIGURE 3:**
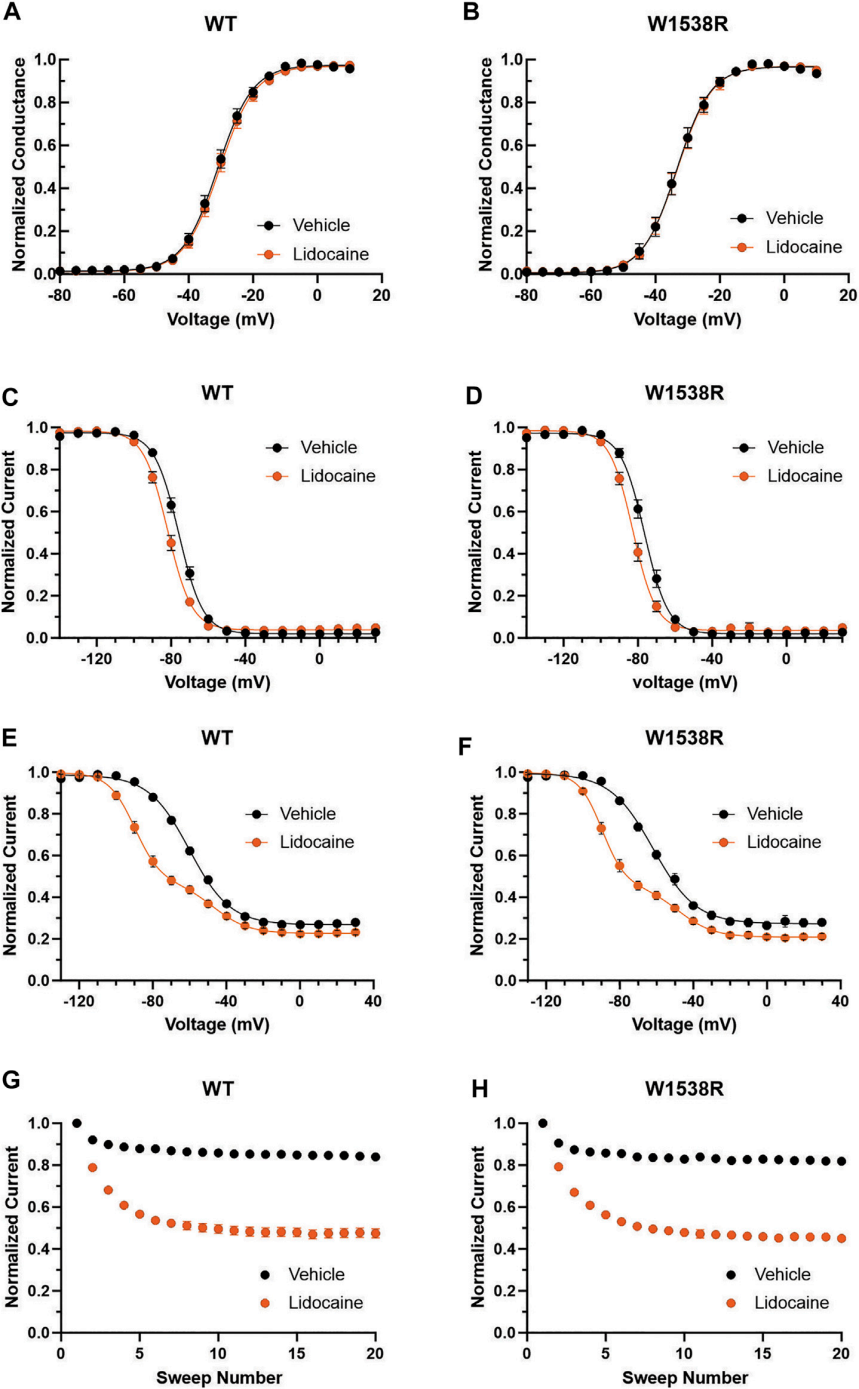
Lidocaine inhibits Na_V_1.7-W1538R mutant channels. The effect of 80 μM lidocaine on the voltage-dependence of activation, shown with single (vehicle) and double (lidocaine) Boltzmann fits of normalized conductance transformed from the current-voltage plot peak inward currents, of **(A)** Na_V_1.7-WT was indiscernible from **(B)** Na_V_1.7-W1538R channels. **(C,D)** Relative to vehicle (extracellular bath solution, black), lidocaine (orange) hyperpolarizes the voltage-dependence of fast-inactivation of both Na_V_1.7-WT and Na_V_1.7-W1538R. **(E,F)** Lidocaine additionally hyperpolarizes the voltage-dependence of slow-inactivation of both Na_V_1.7-WT and Na_V_1.7-W1538R. **(G,H)** Lidocaine also enhances the use-dependent inhibition of Na_V_1.7-WT and Na_V_1.7-W1538R.

The W1538R mutation, which maps to the VSD4, has been shown to completely abolish the effect of lacosamide on Na_V_1.7 gating mechanisms at therapeutic concentrations (Labau et al., 2020). Whether this mutation exerts an allosteric effect on the LA site in the pore to reduce the effect of lacosamide remains unclear. To test whether W1538R impairs the LA binding site in Na_V_1.7, we assessed the blocking effect of lidocaine on Na_V_1.7-W1538R channels (Figure 3) using the Qube automated electrophysiology platform and analyzed the data with unpaired Student’s t-tests. In response to 80 μM lidocaine, and in line with previous studies (Chevrier et al., 2004), lidocaine did not evoke a shift in the voltage-dependence of activation of Na_V_1.7-WT channels (Figure 3A). Likewise, W1538R channel activation was unaffected by the presence of the drug (Figure 3B). As expected from past reports (Chevrier et al., 2004; Sheets et al., 2011; Wang et al., 2015), lidocaine significantly hyperpolarized the WT channel voltage-dependence of both fast-inactivation (Figure 3C) and slow inactivation (Figure 3E). Similar results were observed in the mutant channels where lidocaine significantly hyperpolarized the V_1/2_ of fast-inactivation (Figure 3D) and slow-inactivation of W1538R channels (Figure 3F). Lidocaine induced a two-component slow inactivation shift in both variants, and was fitted to a double Boltzmann. Finally, lidocaine significantly enhanced the use-dependence of inhibition in both WT (Figure 3G) and W1538R channels (Figure 3H), indicating that the effect of lidocaine was not affected by the presence of the W1538R mutation. The activation and inactivation V_1/2_s as well as the degree of block of both variants are listed in Table 2.

**TABLE 2:** **-**Inhibitory effects of sodium channel blockers Lidocaine (LDC) and ICA-121431 on NaV1.7-WT and W1538R mutant channels. Lidocaine blocked both Na_V_1.7 WT and W1538R channels, by hyperpolarizing fast and slow inactivation as well as increasing use-dependence inhibition. Na_V_1.3-selective blocker ICA-121431 potently inhibited Na_V_1.7-W1538R mutant channels while having no effect on Na_V_1.7-WT channels. Treatment with ICA-121431 caused a hyperpolarizing shift in both fast and slow inactivation and increased use-dependent block of W1538R mutant channels. Like for 300 μM lacosamide, 80 μM lidocaine-induced shift in slow inactivation of both Na_v_1.7-WT and W1538R channels was best fitted by a double Boltzmann, hence the first V_1/2_ of the fit is reported in the table below, while vehicle was best fitted to a single Boltzmann. Data are presented as Means ± SEM.

		—	Nav1.7-WT	Nav1.7-W1538R
Lidocaine	Conductance V_1/2_ (mV)	Vehicle	−30.83 ± 0.98, *n* = 24	−33.08 ± 1.19, *n* = 18
		LDC	−30.02 ± 0.98, *n* = 24, *p* = 0.44	−32.6 ± 1.16, *n* = 18, *p* = 0.55
		80 μM		
	Fast inactivation V_1/2_ (mV)	Vehicle	−75.93 ± 0.92, *n* = 38	−76.54 ± 1.28, *n* = 19
		LDC	−81.73 ± 0.94, *n* = 38, *p* = 6.71e-13	−82.59 ± 1.13, *n* = 19, *p* = 1.03e-9
		80 μM		
	Slow inactivation	Vehicle	−60.20 ± 0.87, *n* = 35	−61.48 ± 1.08, *n* = 19
	V_1/2_ (mV)	LDC	−89.92 ± 0.49	−89.63 ± 0.34
		80 μM	*N* = 35	*N* = 19
			*p* = 6.82e-26	*p* = 5.73e-19
	Use-dependence of inhibition	Vehicle	0.84 ± 0.008, *n* = 23	0.82 ± 0.01, *n* = 14
		LDC	0.48 ± 0.02, *n* = 23, *p* = 1.16e-7	0.45 ± 0.02, *n* = 14, *p* = 3.70e-5
		80 μM		
ICA-121431	Activation	Vehicle (0.1% DMSO)	−30.35 ± 1.74, n = 13	−31.48 ± 1.72, *n* = 11
	V_1/2_ (mV)	ICA (0.1 μM)	−32.32 ± 1.90, *n* = 9, *p* = 0.46	−31.64 ± 1.1, *n* = 14, *p* = 0.94
	Fast inactivation	Vehicle (0.1% DMSO)	−74.13 ± 1.89, *n* = 12	−72.99 ± 0.95, *n* = 27
	V_1/2_ (mV)	ICA (0.1 μM)	−74.83 ± 2.07, *n* = 12, *p* = 0.81	−77.02 ± 0.99, *n* = 22, *p* = 0.0054
	Slow inactivation	Vehicle (0.1% DMSO)	−57.66 ± 1.8, *n* = 12	−57.25 ± 1.2, *n* = 26
	V_1/2_ (mV)	ICA (0.1 μM)	−55.34 ± 2.3, *n* = 9, *p* = 0.81	−66.37 ± 1.5, *n* = 24, *p* < 0.0001
	Use-dependence of inhibition	Vehicle (0.1% DMSO)	0.88 ± 0.01, *n* = 19	0.92 ± 0.009, *n* = 30
		ICA (0.1 μM)	0.88 ± 0.01, *n* = 19, *p* = 0.92	0.7 ± 0.03, *n* = 38, *p* < 0.0001

### 3.4 At 10-Fold the Clinical Dose, Lacosamide Recovers its Inhibitory Properties of W1538R Mutant Channels

**FIGURE 4:**
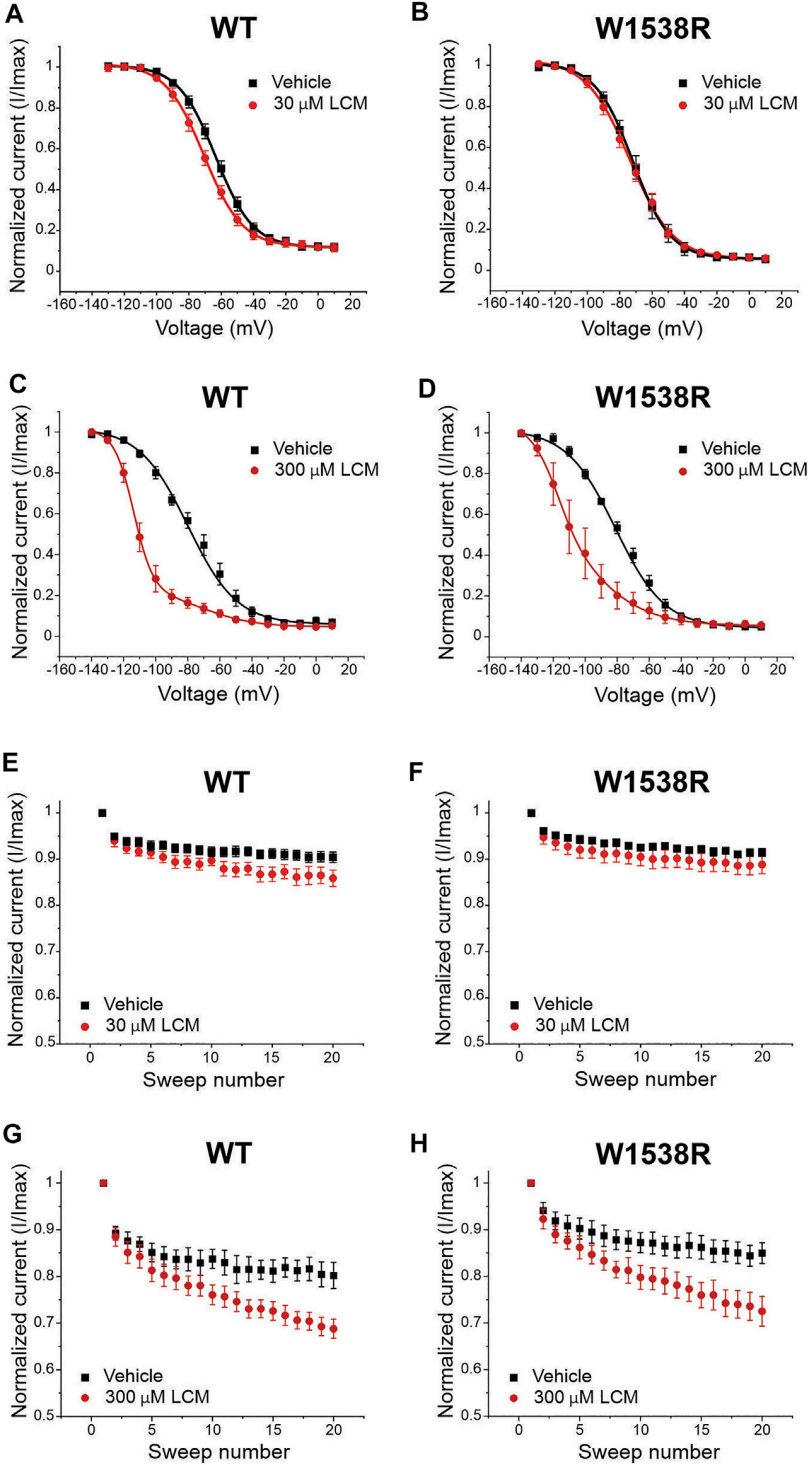
Na_V_1.7-W1538R channels are inhibited after a 10-fold increase in the lacosamide therapeutic concentration. The effect of lacosamide on Na_V_1.7 voltage-gating properties were evaluated for **(A–D)** Voltage-dependence of slow inactivation and **(E–H)** use-dependence of inhibition at 20 Hz. The slow inactivation curves were fitted to a single Boltzmann for vehicle and 30 μM lacosamide, and to a double Boltzmann equation for 300 μM lacosamide. Cells expressing Na_V_1.7-WT or Na_V_1.7-W1538R were either treated with lacosamide at the clinically-achievable dose of 30 μM (shown in red, **A,B,E,G**), its 10-fold increase of 300 μM (shown in dark red, **C,D,F,H)**, or vehicle (black; extracellular bath solution).

Lacosamide’s poor efficacy in blocking W1538R mutant channels, whether in heterologous cell models (Labau et al., 2020) or in human carriers (de Greef et al., 2019), has thus far exclusively been studied at the clinically-achievable dose of 30 μM. To further evaluate the affinity profile of the W1538 residue as a binding site for lacosamide, we investigated whether a 10-fold increase in lacosamide (300 μM) restores the drug-induced block of Na_V_1.7-W1538R channels. As expected, lacosamide had no effect on the voltage-dependence of activation or steady-state fast-inactivation in either Na_V_1.7-WT or W1538R channels at either 30 or 300 μM (*Data not shown*), using unpaired Student’s t-tests. In an attempt to directly compare our past results using 30 μM lacosamide (Labau et al., 2020) to the gating outcomes from a 10-fold increase in lacosamide concentration, we first reproduced the significant hyperpolarizing shift observed in the V_1/2_ of slow-inactivation of Na_V_1.7-WT channels exposed to 30 μM lacosamide (Figure 4A) as well as the enhanced use-dependence of inhibition at 20 Hz (Figure 4E). In WT channels, 300 μM lacosamide induced a two-component slow inactivation shift and increased use-dependent block (Figures 4C,G). We also reproduced the lack of effect of 30 μM lacosamide on W1538R channel’s V_1/2_ of slow-inactivation (Figure 4B) and use-dependence of inhibition (Figure 4F). However, a 10-fold increase in the concentration of lacosamide significantly blocked W1538R channels by hyperpolarizing the V_1/2_ of the first phase of two-component slow-inactivation (Figure 4D) and increased the use-dependence of inhibition of the mutant channel (Figure 4H), indicating that higher concentrations rescue the inhibitory action of lacosamide on the Na_V_1.7-W1538R mutant channels. Furthermore, the two phase slow inactivation shift is suggested to be evoked by higher concentrations of lacosamide (Figures 4E,F) and lidocaine (Figures 3E,F), where the effect of the compounds are primarily on the more hyperpolarized component. We speculate that while a fraction of channels does not change in the presence of the compound, the majority does. Therefore, the resulting shift in slow inactivation whether fitted to a single or double-Boltzmann remains significant compared to vehicle. Also, the large hyperpolarization of slow-inactivation of WT and W1538R subjected to 300 μM lacosamide, initiated at voltages as low as -130 mV, and in the absence of a preceding plateau phase, might suggest that the magnitude of the shift may be even larger than calculated in Figures 4C,D. The datasets are summarized in Table 1.

### 3.5 The Mutant W1538R Arginine Residue Confers Na_V_1.7 Channels Responsiveness to ICA-121431

**FIGURE 5:**
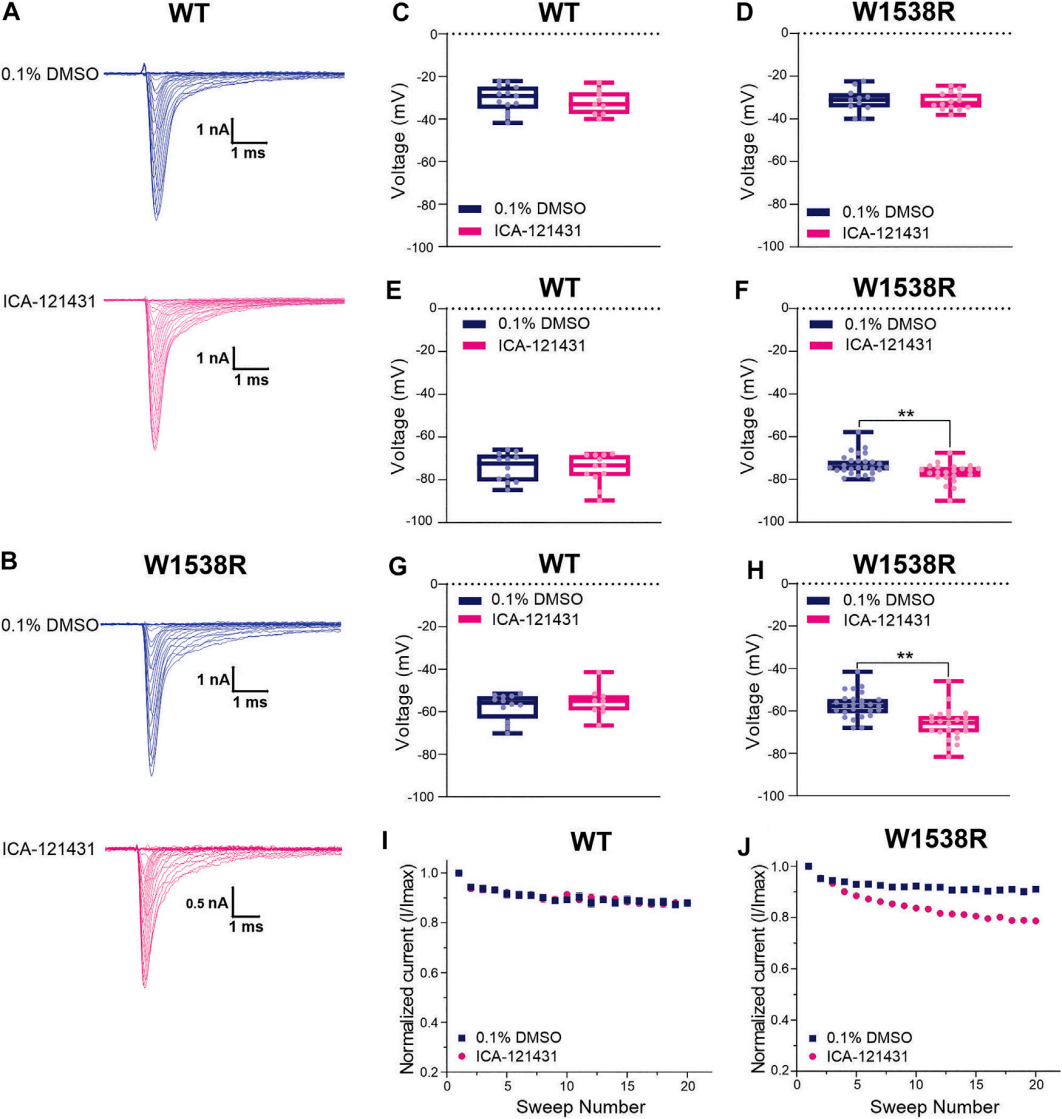
The aryl-sulfonamide Na_V_1.3-selective inhibitor ICA-121431 blocks Na_V_1.7-W1538R mutant channels. The sodium current traces were produced using the activation protocol from Na_V_1.7 **(A)** WT and **(B)** W1538R-expressing cells. The half-inactivation voltage (V_1/2_) of **(C,D)** Activation, **(E,F)** Fast-inactivation and **(G,H)** Slow-inactivation was calculated from the Boltzmann fits and averaged for each genotype in response to 0.1 μM ICA-121431 (pink) or vehicle (0.1% DMSO in extracellular bath solution; blue). Data are shown as scatter plots and boxes at the 25 and 75th percentile of the data with medians ± min/max. P* < 0.05, P** < 0.01. The use-dependence of inhibition of **(I)** WT and the **(J)** W1538R channels in response to treatment with 0.1 μM ICA-121431 or vehicle was normalized to the first pulse and averaged across a series of twenty 20 Hz pulses. Data are shown means ± SEM. p* < 0.05, p** < 0.01.

The arginine (R1538) residue in the mutant Na_V_1.7-W1538R channel has been shown to contribute to Na_V_1.7 aryl sulfonamide inhibitor selectivity and is the naturally occurring residue present in Na_V_1.3 (R1560) channels (McCormack et al., 2013), which may contribute to the resistance of Na_V_1.3 to lacosamide inhibition, compared to Na_V_1.7 channels (Sheets et al., 2008). Here, we first tested whether the W1538R mutation alone is sufficient to confer Na_V_1.7 channels responsiveness to ICA-121431, using the Qube and unpaired Student’s t-test analysis. The selectivity of 0.1 μM ICA-121431 was confirmed by the absence of effect on Na_V_1.7-WT channels for any of the studied parameters (Figures 5A,C,E,G,I). The compound had also no effect on the voltage-dependence of activation, measured as the conductance V_1/2,_ of W1538R channels (Figure 5D) in comparison to vehicle (0.1% DMSO). However, it did significantly shift W1538R fast-inactivation (Figure 5F) as well as hyperpolarized the slow-inactivation V_1/2_ by nearly 10 mV (Figure 5H), compared to vehicle. Furthermore, ICA-121431 significantly enhanced the W1538R channel use-dependence of inhibition (Figure 5J), indicating that the arginine residue found in the W1538R mutant confers sensitivity to ICA-121431 blocker on Na_V_1.7 channels. The respective V_1/2_s and degree of block of each Na_V_1.3 variant in response to ICA-121431 are reported in Table 2.

### 3.6 The Na_V_1.3-R1560W Mutation Does not Confer Lacosamide Sensitivity to the Channel

Since the W1538R mutation abolishes the effect of 30 μM lacosamide on Na_V_1.7, we investigated whether the naturally occurring Na_V_1.7-W1538 tryptophan residue contributes to differences in lacosamide’s subtype selectivity. Using automated patch-clamping on the Qube and One-Way ANOVA with Dunnett’s multiple comparison analysis, we tested the effect of 30 and 300 μM lacosamide on mutant Na_V_1.3 channels in which the arginine residue (R1560) is substituted with a tryptophan, R1560W, to determine whether the presence of a tryptophan residue in Na_V_1.3 can confer sensitivity to lacosamide.

**FIGURE 6:**
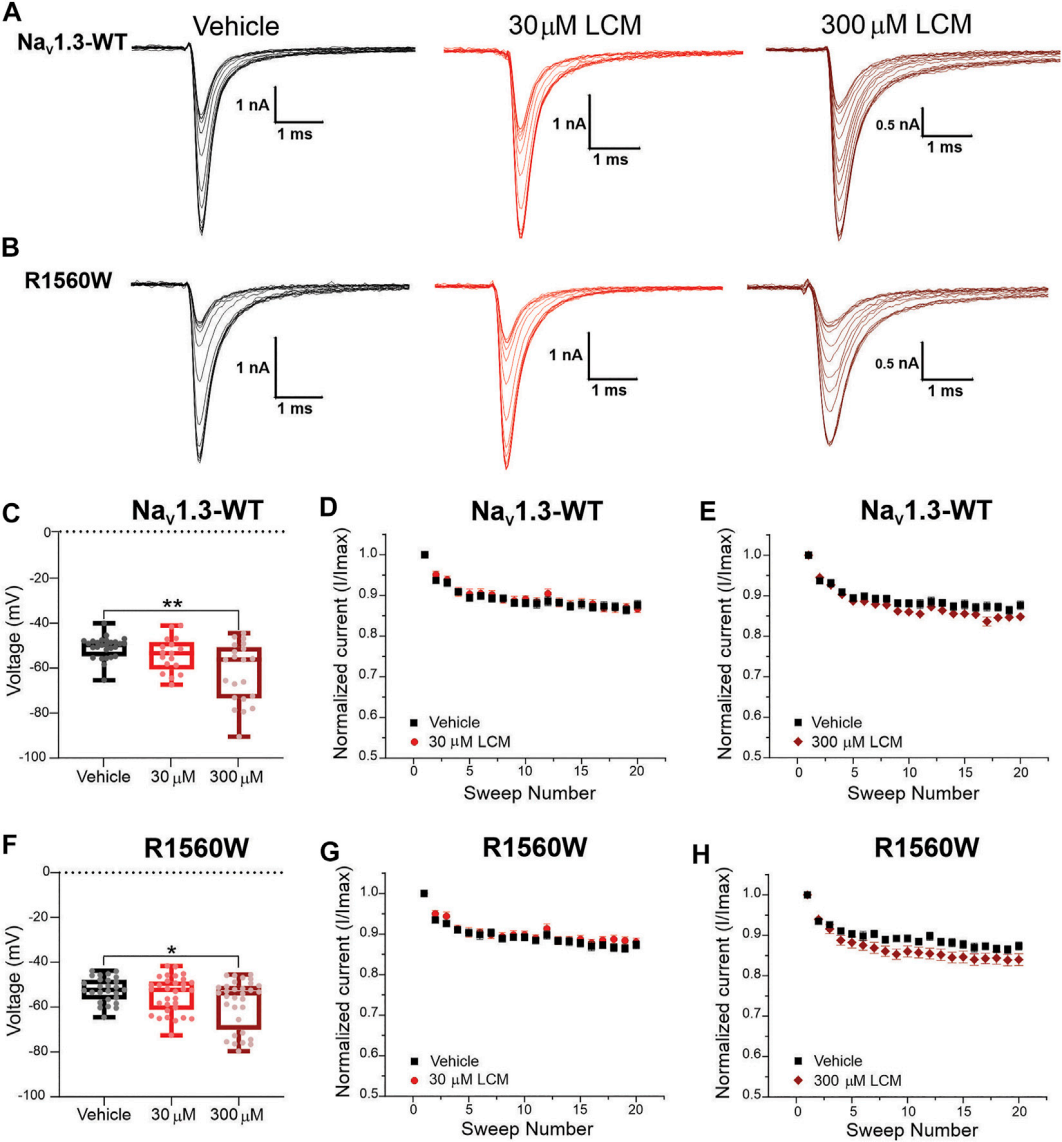
The Na_V_1.3-R1560W mutation in is not sufficient to confer lacosamide sensitivity to Na_V_1.3 channels. The R1560 residue in Na_V_1.3, which corresponds to the W1538R mutation in Na_V_1.7, was mutated from an arginine (R) to a tryptophan (W) to match the sequence of Na_V_1.7 WT at this site, and the responsiveness of the Na_V_1.3-R1560W to lacosamide. **(A,B)** Representative sodium current traces were generated from the activation protocol and show Na_v_1.3-WT **(A)** and Na_v_1.3-R1560W **(B)** currents in response to vehicle (black), 30 μM (red) and 300 μM (dark red) lacosamide. Dose-dependent effects of 30 and 300 μM lacosamide were measured for **(C–F)** slow-inactivation and **(D,E,G,H)** use-dependence of inhibition at 20 Hz for human Na_V_1.3-WT and Na_V_1.3-R1560W channels using an automated electrophysiology platform (Qube 384, Sophion Bioscience) **(C–F)** Data are shown as individual scattered points and boxes at the 25 and 75th percentile of the data with medians ± min/max. Significance was evaluated with One-way ANOVA. p* < 0.05, p** < 0.01. **(C–F)** Data are shown as the averaged current normalized to the first pulse.

At 30 μM, lacosamide failed to shift the voltage-dependence of slow-inactivation in both Na_V_1.3-WT (Figure 6C) and Na_V_1.3-R1560W channels (Figure 6F) compared to vehicle. Likewise, use-dependence of inhibition at 20 Hz was unaltered by the presence of 30 μM lacosamide in either variant (Figures 6D,G), demonstrating that a single amino acid substitution in Na_V_1.3 channels cannot bestow pharmacological responsiveness to this therapeutic concentration of lacosamide. However, at a 10-fold increase in lacosamide concentration, the voltage-dependence of slow-inactivation of Na_V_1.3-WT channels was hyperpolarized by 11.5 mV (Figure 6C), in the same range as Na_V_1.7-WT at 30 μM (Figure 4A), and by 6.2 mV in the R1560W channels (Figure 6F) compared to vehicle. However, use-dependence of inhibition was not increased at 300 μM for either Na_V_1.3-WT (Figure 6E) or R1560W (Figure 6H) channels from control, which is in contrast to our finding that 300 μM lacosamide significantly increased Nav1.7-W1538R use-dependence of inhibition (Figure 4H). These results suggest that the R1560 residue in Na_V_1.3 may contribute to the sodium channel isoform sensitivity to lacosamide, but that it is not sufficient to explain the subtype differences in lacosamide responsiveness. The slow inactivation and use-dependence values are reported in Table 1.

## 4 Discussion

The mechanisms by which lacosamide inhibits sodium channels at clinically-achievable concentrations in an isoform-dependent manner, compared to other inhibitors in the pharmacopoeia, have long been debated and are not well understood. Existing data suggest that residues outside the pore can affect channel sensitivity to lacosamide and raise the possibility that lacosamide’s effect may also be dependent on interactions with both the VSD4 and the LA binding site in the pore-forming region of VGSCs. In the present study, we describe a new pathway for lacosamide’s mechanism of action, where lacosamide requires the W1538 residue in VSD4 and an intact LA binding site to block Na_V_1.7 at the clinically-achievable concentration of 30 μM. The presence of an arginine residue in Na_V_1.7-W1538R channels does not abolish LA pore access and confers sensitivity to aryl-sulfonamide blocker ICA-121431. Conversely, the W1538 tryptophan residue does not explain lacosamide’s resistance in other isoforms, specifically in Na_V_1.3 channels. Taken together, our results highlight the importance of the W1538 residue combined with pore accessibility in allowing 30 μM lacosamide’s inhibitory effects, and suggest the contribution of W1538 for VGSC isoform-specificity.

*In silico* docking analysis in human Na_V_1.7 channels predicted a pocket that includes W1538 as the most energetically-favorable binding site to lacosamide, closely followed by the LA site in the pore (Figure 1), suggesting a novel mechanism for the drug to access the pore. Previous molecular docking studies that have described lacosamide as a pore-binding ligand only studied the compound in the homotetramer bacterial VGSCs (Tikhonov and Zhorov, 2017). Here, we provide a new hypothesis in which both W1538 and F1737/Y1744 are necessary for lacosamide’s inhibitory effect of the mammalian Na_V_1.7 channel, consistent with the prediction that lacosamide efficiently binds at both sites (Figures 1C,D). Furthermore, the structural distance between the two regions indicate that W1538R is unlikely to mechanically interfere with pore binding (Figures 1A,B), but might impact the ability of lacosamide to utilize the nearby fenestration to reach the pore. While this in silico docking method neither allows the application of atomic forces nor considers interactions with β subunits, the model points to W1538 as a strong putative binding site for lacosamide and, combined with our previous findings (Labau et al., 2020), indicates that the W1538 residue is necessary to enable lacosamide’s inhibition of Na_V_1.7 channels.

Consistent with previous studies (Jo and Bean, 2017; Wang and Wang, 2014)**,** we demonstrate that lacosamide interacts with the LA site, or at least requires the F1737 and Y1744 residues to exert its inhibitory effect on Na_V_1.7 channels (Figure 2). These results suggest that lacosamide’s mechanism of action aligns with the hypothesis that binding to the LA site may correlate with preferential binding to the fast-inactivated state but with very slow kinetics (Jo and Bean, 2017). However, while an intact LA site in the pore is necessary for lacosamide’s effect, it is not sufficient to explain the striking differences in gating induced by lacosamide compared to other pore-binding agents that modulate fast inactivation, nor the lack of drug sensitivity observed in Nav1.7-W1538R channels (Labau et al., 2020). The requirement for an interaction of lacosamide with W1538 in VSD4 before reaching the pore might underlie the slow kinetics of binding to the fast-inactivated state of Na_V_1.7 channels that have been previously reported (Jo and Bean, 2017).

The W1538R mutation might block pore access to lacosamide or cause an allosteric effect that prevents conformational changes exposing the LA binding site in the pore where the drug needs to bind to cause its inhibitory effect. During activation, the DIV S6 segment undergoes a spatial re-arrangement from a closed to an open configuration that exposes the previously hidden F1737 residue of the LA site via asynchronous voltage sensor movements (Goldschen-Ohm et al., 2013; Pless et al., 2011). However, the W1538R mutation did not prevent pore binding of lidocaine and inhibition of Na_V_1.7 channels (80 μM, Figure 3). This finding aligns with a previous report showing that W1538R, together with Na_V_1.7-Y1537S and Na_V_1.7-D1586E, had no effect on the LA tetracaine-induced Na_V_1.7 channel inhibition (McCormack et al., 2013). Therefore, W1538R is unlikely to prevent a conformational shift in the pore that is needed for lacosamide’s inhibitory effect on VGSCs. Nonetheless, since 30 μM lacosamide does not inhibit Nav1.7-W1538R channels (Labau et al., 2020) (Figures 4A,B, Figures 4E-F), this residue might selectively regulate the access of lacosamide but not LAs to the pore.

We further explored the role of W1538 in lacosamide inhibition of Na_V_1.7 channels and tested the effect of a 10-fold increase in lacosamide concentration (Figure 4). We show that, at 300 μM, the W1538R-induced lacosamide block can be overcome, suggesting that the likelihood of lacosamide access to the pore is increased and is sufficient to rescue the inhibitory effect on the channel. This phenomenon may be explained by an allosteric W1538R-induced change in binding affinity at the pore, whereby higher concentrations of lacosamide can circumvent the need for W1538 facilitation and bind to the pore. Otherwise, the W1538 residue (alone or together with other sites) might be viewed as an initial binding site of lacosamide which may impose the slow phase of the drug’s interaction with the channel, allowing an increase in the local effective concentration of the drug in the vicinity of the pore. The next phase may correspond to lacosamide’s fast binding phase, which has been suggested to correlate with binding to partially open channels, with rapid access to the non-obstructed inner pore (Jo and Bean, 2017). These data support a role for W1538 in the slow phase at a clinically-achievable concentration. However, at 10x higher concentrations, alternative pathways may be used, where lacosamide no longer requires the integrity of W1538 to reach the pore and exert its functions.

**FIGURE 7:**
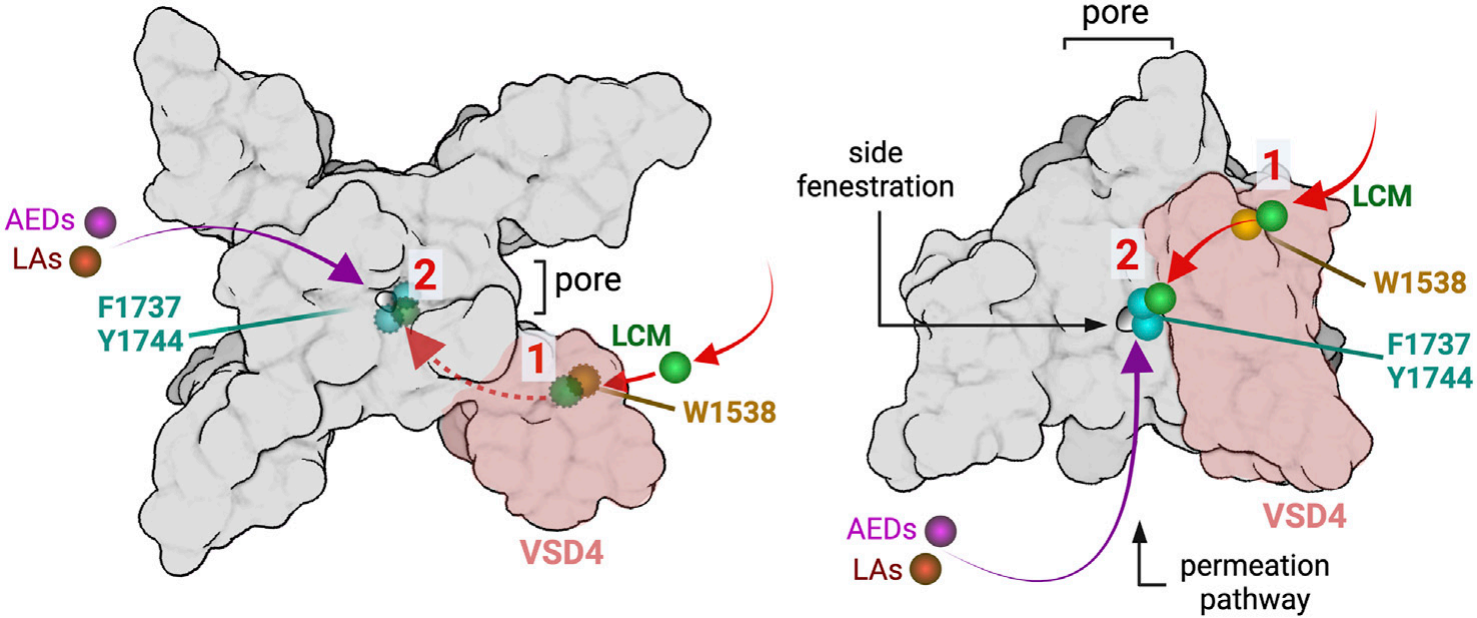
Working hypotheses of lacosamide binding mechanisms. Schematic representation of the Na_V_1.7 channel showing lacosamide (LCM; green) binding residues W1538 (shown in gold) and LA binding pore residues F1737 and Y1444 (shown in cyan). The left panel shows the Na_V_1.7 channel visualized from the top, where, in order to reach the pore, lacosamide may first bind to the W1538 region in VSD4 (shaded in pink), before accessing the pore through the side fenestration where it exerts its inhibitory effects (not visible from the top, visualizable with dotted lines). This process would differ from typical AEDs (magenta) and LAs (red) that can reach the pore without an interaction with the W1538 residue. Alternatively, the W1538 residue might represent an allosteric site modulating lacosamide binding affinity to F1737 and Y1444 in the pore. After coming into contact with VSD4 in the W1538 vicinity, lacosamide is able to access the pore and tightly bind to LA-binding residues. The right panel represents the Na_V_1.7 channel from the side, top down, and shows the suggested path lacosamide may take through the pore’s side fenestration *via* the W1538 residue or directly to the pore after some molecules become W1538-bound.

Lacosamide’s inhibition shares features of AED/LA pore-blocking drugs and the novel aryl sulfonamide blockers. The high state-dependence, the ultra-slow on-rate binding and the role of the W1538 residue in this process are features in common between VSD4 blockers (ie. ICA-121431, PF-05089771) and lacosamide, and the dependence of lacosamide’s inhibition on the LA site in the pore is common with AEDs and LAs (McKerrall and Sutherlin, 2018). VSD4-blockers have been suggested to undergo reorientation steps and to use transitional sites, including pockets that are proximal to the S6 segment, en-route to their final position on VSD4 where they interact with the gating charges in the S4 to effect their inhibitory action, which presumably contribute to their slow rate of channel inhibition (Corry, 2018). Our results suggest a working model in which lacosamide may first bind with high affinity to the W1538 residue in a pre-open state, before transiting to the LA binding site upon channel opening where it blocks the pore. Alternatively, the W1538 motif might act as an allosteric site modulating the binding affinity of lacosamide at the LA/AED pore sites F1737 and Y1444 (Figure 7). It is not clear whether lacosamide’s first interaction with the W1538 residue or its transit to the pore causes the slow on-rate of the drug. Irrespective of this, it appears that lacosamide’s effect on Na_V_1.7 channels follows a hybrid model of interaction with VSD4 and the pore which makes it distinct from both VSD4-binders and pore-blocker inhibitors of VGSCs. Of note, Na_V_1.7 blockers have recently been suggested to have a higher analgesic potential when acting centrally (MacDonald, et al., 2021), a proposal that contradicts efforts to develop selective compounds, such aryl sulfonamides, that are relatively CNS-impermeant. Nevertheless, while lacosamide shares features with VSD4 blockers, it is not a peripherally-restricted drug.

To further understand the similarity between lacosamide and VSD4 blockers, we assessed the role of W1538 in the response of Na_V_1.7 channels to these blockers. We demonstrate that the W1538R mutation is sufficient to render Na_V_1.7 channels responsive to selective Na_V_1.1/Na_V_1.3 blocker ICA-121431 (Figure 5). Our results align with previous studies showing that the W1538 residue is critical in determining VGSC sensitivity to aryl sulfonamide-based blockers (McCormack et al., 2013) and show that this substitution alone leads to a significant ICA-121431-induced block. However, we did not observe that the corresponding substitution in Na_V_1.3 (R1650W) renders these channels sensitive to lacosamide at therapeutic concentrations (Figure 6). These data suggest that the role of this tryptophan residue in lacosamide-induced inhibition at low concentrations appears to be isoform-dependent.

Importantly, W1538, as a moderately conserved residue, is unlikely to be the sole contributor to lacosamide’s VGSC isoform selectivity. Na_V_1.1, Na_V_1.3, Na_V_1.5 and Na_V_1.8 channels might be expected to be less sensitive to lacosamide because they carry different residues at this position (Corry, 2018). Evidence of lacosamide’s weak inhibition of Na_V_1.3 and Na_V_1.5 is well-documented (Sheets et al., 2008; Wang and Wang, 2014). Interestingly, the fact that Na_V_1.3-R1650W channels are not inhibited by 30 μM lacosamide, and the ability to rescue lacosamide inhibition of resistant channels at 300 μM of Na_V_1.7-W1538R (Figure 4), and both Na_V_1.3 (Figure 6) and Na_V_1.5 channels (Sheets et al., 2008; Wang and Wang, 2014), suggests a different pathway for lacosamide to access the pore in the more resistant channel isoforms.

In conclusion, this study presents the W1538 residue as necessary for the initial binding of lacosamide to the Na_V_1.7 channel, facilitating its access to the channel’s pore at therapeutic concentrations. The mechanistic differences between lacosamide, LAs, and conventional AEDs may be explained by the W1538 residue’s participation in lacosamide’s slow binding. Furthermore, our results suggest that while the W1538 residue is necessary to confer lacosamide sensitivity on Na_V_1.7 channels, it is not sufficient to explain the molecular basis for lacosamide-resistant isoforms, suggesting the presence of additional molecular determinants for the drug sensitivity. A better understanding of the hybrid mechanism of inhibition of Na_V_1.7 by therapeutically-achievable concentrations of lacosamide might lead to more selective and effective VGSC blockers.

## Data Availability

The original contributions presented in the study are included in the article, further inquiries can be directed to the corresponding authors.

## Abbreviations

AEDs, Anti-epileptic drugs; GFP, Green fluorescent protein; HEK293, Human embryonic kidney 293; LAs, Local anesthetics; VGSCs, Voltage-gated sodium channels; VSD, Voltage-sensing domain; WT, Wild-type.
